# Supplementary material for: Meta-Analysis and MaxEnt Model Prediction of the Distribution of Phenacoccus solenopsis Tinsley in China under the Context of Climate Change
Source: Insects. 2024 Sep 6;15(9):675. doi: 10.3390/insects15090675 (PMC11432275; doi:10.3390/insects15090675)
Supplement: Supplementary file 1 [file insects-15-00675-s001.zip › insects-3186856-supplementary.pdf]

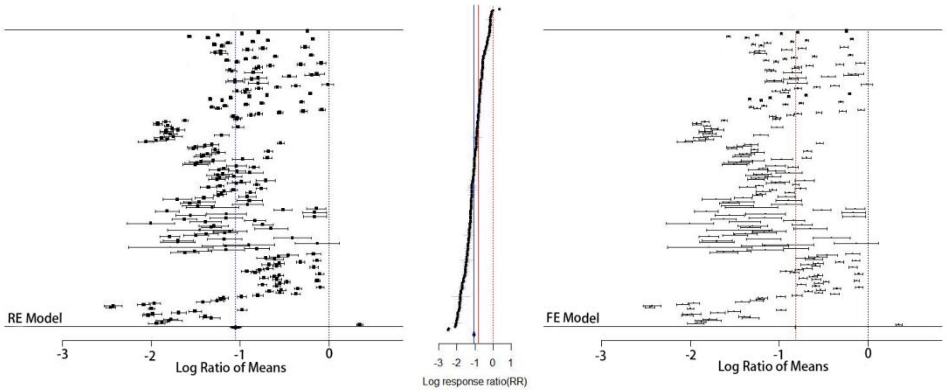

Figure S1 Comparison of effect amount of random effect model and fixed effect model (the left figure shows the calculation result of random effect model, the estimate is -1.0499, the upper and lower 95% confidence interval is -1.1195 to -0.9803, Q=134684.2678, df=220, p<0.0001, I^2=99.87%. The figure on the right shows the calculation results of the fixed effect model. The estimate is -0.8187, the upper and lower 95% confidence interval is -0.8212 to -0.8162, Q=134684.2678, df=220, p<0.0001, and I^2 =99.84%. The calculation results show that under the calculation conditions of random effect model or fixed effect model, with the increase of temperature, the development cycle of *P. Tinsley* is significantly reduced)

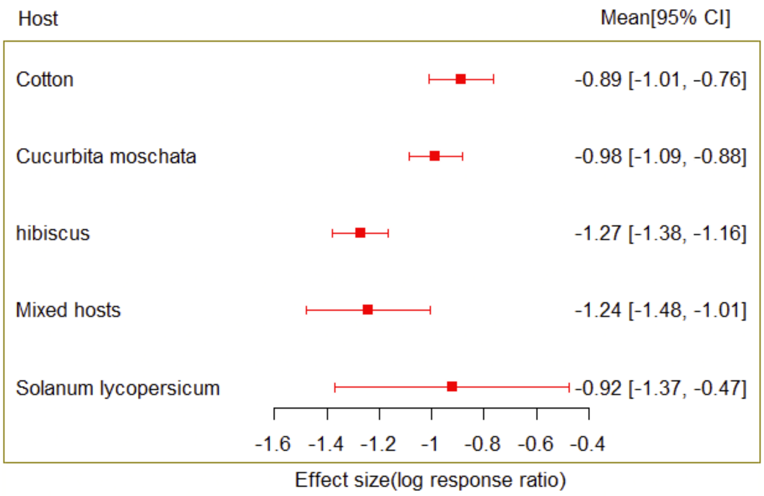

Figure S2 Effect of host on temperature rise test results (Q_m=49.0471, df=4,

p<0.0001)

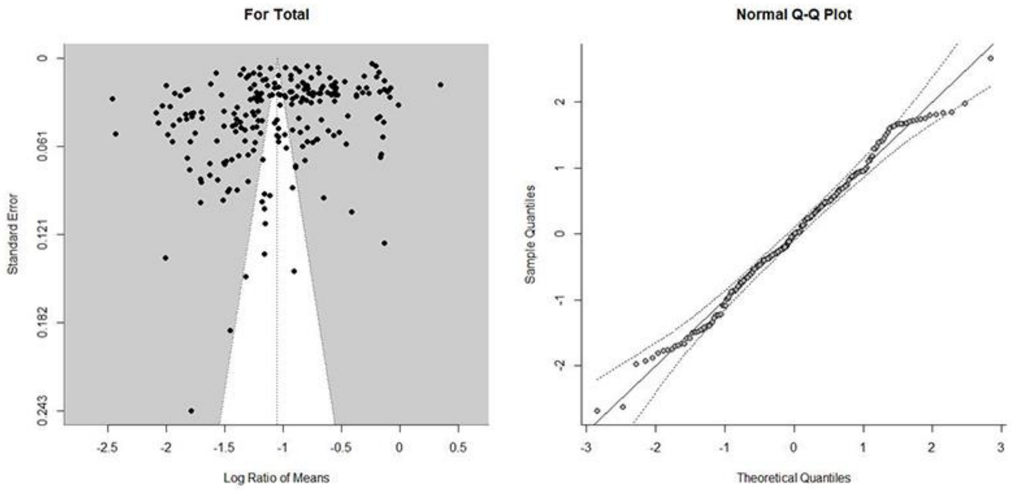

Figure S3 Funnel diagram and q-q diagram are used to check the reliability of the results.
